# Sugarcane Wax Metabolites and Their Toxicity to Silkworms

**DOI:** 10.3390/life13020286

**Published:** 2023-01-19

**Authors:** Li Ma, Mingzheng Duan, Ziwei He, Yu Zhang, Yiting Chen, Bo Li, Muhammad Junaid Rao, Lihua Hu, Lingqiang Wang

**Affiliations:** 1 State Key Laboratory for Conservation and Utilization of Subtropical Agro-Bioresources, College of Agriculture, Guangxi University, 100 Daxue Rd., Nanning 530004, China; 2 Guangxi Key Laboratory of Sugarcane Biology, Guangxi University, 100 Daxue Rd., Nanning 530004, China; 3 College of Life Science and Technology, Guangxi University, Nanning 530004, China

**Keywords:** insecticide, sugarcane wax, metabolome, metabarcoding, silkworm model, 16s rRNA

## Abstract

Sugarcane wax has the potential to be utilized as a novel natural insecticide, which could help to reduce the large yield losses caused by agricultural pests. By employing the gas chromatography–mass spectrometry (GC-MS) approach, we conducted a study to analyze the composition of epicuticular wax from the rind of the sugarcane variety YT71210. A total of 157 metabolites, categorized into 15 classes, were identified, with naphthalene, a metabolite with insect-resistant properties, being the most prevalent. The feeding trial experiment suggested that sugarcane wax is toxic to silkworms by impacting the internal organs. Intestinal microbial diversity analysis suggested that the abundance of *Enterococcus* genus was significantly increased in both ordure and gut of silkworm after wax treatment. The results indicated that the feeding of wax has an adverse effect on the gut microbial composition of silkworms. Our findings lay a foundation for the efficacy of sugarcane waxes as a valuable natural insecticide and for the prediction of promising sugarcane varieties with insect resistance.

## 1. Introduction

Sugarcane (*Saccharum* spp.) is a major industrial crop mainly grown in tropical or subtropical regions across the world, such as Brazil, China and India [1,2]. Sugarcane has extensive values in food, industry, agriculture and medical applications. It is cultivated mainly for sugar production and supplies >70% of the world’s sugar [2]. It has gained increased global prominence because of its superior potential for use as a sustainable and renewable source of biomass and bioenergy in the last 20 years [3]. Additionally, bagasse has also been used as a potential soil amendment and improve soil health and fertility level [4,5]. As for medical value, many studies have reported that sugarcane is the source of many high-value medicinal metabolites, such as one recently by Rao (2022), who characterized many amino acids metabolites with antioxidant capacity form sugarcane rinds [6]. Therefore, sugarcane is a resource worth being continuously explored and developed.

Interestingly, some sugarcane varieties produce a large amount of visible epicuticular wax on the surface of the rind and leaf sheath. However, few studies have been reported on the composition, biosynthesis and biological function of the epicuticular wax of sugarcane. Agricultural pests cause significant yield loss, and insecticide-resistant pests become more difficult to control [7]. It is critical to develop natural insecticide with the purpose of reducing chemical pesticide usage. The roles of wax metabolites in the pest resistance of sugarcane and their potential to be developed as biological insecticides remain to be explored.

Several studies have reported that the purified wax from sugarcane rind consists of metabolites of alkanes, esters, n-triacontanol, policosanol and D-003 acids, and has pharmaceutical, agricultural and industrial applications [8,9]. The sugarcane borer, *Datraea sacharalis*, is one of the most serious pests, destroying the plant by invading the young stem portion of the sugarcane [10]. Epicuticular wax may act as a barrier, influencing the behavior and survival of larvae, and this can partially explain the differences between the sugarcane varieties in insect resistance [11]. Furthermore, some wax metabolites, such as alcohols and short-length chain aldehydes, have resistance to stalk borers [12,13]. More recently, Wartha et al. (2022) identified some components of sugarcane epicuticular wax and used these metabolites to classify the genotypes susceptible or resistant to the initial attack of sugarcane borers (*Diatraea saccharalis*) [14]. Therefore, it is of prospect to study the composition of sugarcane epicuticular wax and its effect of insect resistance.

Insect gut symbiotic microbiota play essential roles in the growth, development, pathogenesis and environmental adaptation of host insects [15]. To assess the anti-insect effect of sugarcane wax, setting up a feeding experiment with a model animal is a convenient and effective method. The silkworm (*Bombyx mori*) is a promising model animal for assessing health safety and environmental pollutants in many studies [16,17,18,19,20,21], and it can be an alternative to sugarcane borer (*Diatraea sacharalis*) in feeding trail experiments based on the following reasons. First, the silkworm and sugarcane borer are members of the same order (Lepidoptera) and the silkworm is sensitive to chemical compounds such as pesticides, drugs and heavy metals [22]. Furthermore, the silkworm has been used as a material in many previous studies that have focused on the investigation of the microbial communities in guts with newly developed sequencing technologies [16,17,18,19,20,21]. It was clear that gut-inhabiting microorganisms play an important role in the host’s health, with impacts ranging from improving host metabolism to shaping the immune system [23,24,25]. Analysis of the alteration of the intestinal microbial communities of wax-fed silkworms will assist in understanding the mechanism underlying the insect resistance of sugarcane wax.

With the development of nucleic acid sequencing technology, next-generation sequencing techniques (NGS) have been widely used in the study of insect gut microbial diversity [15]. Among them, the metabarcoding sequencing technology based on NGS could reveal the microbial diversity and taxon of insect gut in detail. E.g., Mancini (2018), estimated bacteria diversity in different organs of mosquito by 16s rRNA metabarcoding sequencing study [26]; Dong (2018) reported differences in silkworm gut microbiota between different diet [20]. These previous studies indicated that metabarcoding sequencing technology could help us to reveal the gut microbial communities of insects. 

In this study, we investigated the composition of epicuticular wax collected from the sugarcane variety YT71210. Gas chromatography–mass spectrometry (GC-MS) was employed to separate, detect, quantify, and identify metabolites in complex extracts of the sugarcane wax. Then, a feeding experiment on silkworm with sugarcane wax was conducted to evaluate the growth performance of the silkworm. Finally, the microbiological process in the guts of wax-fed silkworms was investigated using 16s RNA metabarcoding sequencing.

## 2. Materials and Methods

### 2.1. Materials 

The YT71210 used in this study is a sugarcane variety widely cultivated in South China. The materials were planted at the experimental farm on the campus of Guangxi University, Nanning city, Guangxi, China, in year 2021. Wax samples were gently and quickly scraped from the surface of the +1–+15 internode, from the bottom to the top of YT71210 plants and were immediately stored at −80 °C. At least 3 g of wax was sampled with three biological replicates for metabolome analysis. At least 15 g of wax of variety of YT71210 was used for silkworm feeding experiments. Silkworm variety Liangguang No. 2 was maintained in Guangxi Sericulture Institute, Nanning city, Guangxi, China and was used as model insect in this study. We chose fourth instar larva to begin the experiment’s growth phase.

### 2.2. Methods

#### 2.2.1. Metabolome Analysis

Metabolomics study was conducted by GC–MS. The conditions were as below, samples were ground to powders in liquid nitrogen. To inhibit any enzyme reaction, 1 g (1 mL) of the powder was quickly transferred to a 20 mL head-space vial (Agilent, Palo Alto, CA, USA) containing a NaCl saturated solution. Crimp-top caps with TFE-silicone headspace septa (Agilent) were used to seal the vials. At the time of SPME analysis, each vial was placed in 100 °C for 5 min, then a 120 µm divinylbenzene fibre (Agilent) was exposed to the headspace of the sample for 15 min at 100 °C. Following sampling, desorption of the VOCs from the fiber coating was carried out in the injection port of the GC apparatus (Model 8890; Agilent) at 250 °C for 5 min in splitless mode. The identification and quantification of VOCs were carried out using an Agilent Model 8890 GC and a 5977B mass spectrometer (Agilent), equipped with a 30 m × 0.25 mm × 0.25 μm DB-5MS (5% phenyl-polymethylsiloxane) capillary column. Helium was used as the carrier gas at a linear velocity of 1.0 mL/min. The injector temperature was kept at 250 °C and the detector at 280 °C. The oven temperature was programmed from 40 °C (3.5 min), increasing at 10 °C/min to 100 °C, at 7 °C/min to 180 °C, at 25 °C/min to 280 °C, holding for 5 min. Mass spectra were recorded in electron impact (EI) ionization mode at 70 eV. The quadrupole mass detector, ion source and transfer line temperatures were set, respectively, at 150, 230 and 280 °C. Mass spectra was scanned in the range *m*/*z* 50–500 amu at 1 s intervals. Identification of volatile compounds was achieved by comparing the mass spectra with the data system library (MWGC or NIST) and linear retention index. Volatiles were detected by MetWare (http://www.metware.cn/; accessed on 4 June 2022) based on the Agilent 8890-5977B platform.

#### 2.2.2. Treatment of Feeding Experiment for Silkworm

We raised silkworms under normal conditions to the fourth instar larvae for use in experiments. The wax feeding experimental design is as follows, control group (CK): fresh mulberry leaves were disinfected under UV for 10 min and fed, wax group (WAX): fresh mulberry leaves were disinfected under UV for 10 min and evenly sprinkled with wax powder. Three replicates were set up for each of CK and WAX groups, with 5 silkworms per replicate. The larvae were placed in an incubator at a temperature of (25 ± 1) °C, humidity of 80% ± 5% and a photoperiod of 12 h(L)/12 h(D) for the experiment. Feeding was quantified daily, excreta were collected, and the mass of worms, as well as the number of disease and death, were recorded. Mulberry leaves were collected from Guangxi University campus, Nanning city, Guangxi, China.

Ten days after feeding, the ordure of silkworms were collected as samples from CK and WAX groups, and the silkworms were dissected, and main intestinal tracts were taken out as gut samples and were stored at −80 °C.

#### 16s rRNA Metabarcoding Analysis

As described previously [27], DNA was extracted from 0.1 g of ordure and gut samples using the HiPure Stool DNA Kits (Magen, Guangzhou, China) according to the manufacturer’s protocol. The 16S rDNA V3–V4 region of the ribosomal RNA gene was amplified by PCR (95 °C for 2 min, followed by 27 cycles at 98 °C for 10 s, 62 °C for 30 s, and 68 °C for 30 s and a final extension at 68 °C for 10 min) using primers 341F (CCTACGGGNGGCWGCAG) and 806R (GGACTACHVGGGTATCTAAT) for bacteria [28], where the barcode is an eight-base sequence unique to each sample. PCR reactions were performed in triplicate 50 μL mixture containing 5 μL of 10 × KOD Buffer, 5 μL of 2.5 mM dNTPs, 1.5 μL of each primer (5 μM), 1 μL of KOD Polymerase, and 100 ng of template DNA. Then, amplicons were extracted from 2% agarose gels and purified using the AxyPrep DNA Gel Extraction Kit (Axygen Biosciences, Union City, CA, USA) according to the manufacturer’s instructions and quantified using ABI StepOnePlus Real-Time PCR System (Life Technologies, Foster City, CA, USA). Purified amplicons were pooled in equimolar and paired-end sequenced (2 × 250) (PE250) on an Illumina platform (Novaseq 6000 sequencing) according to the standard protocol.

For the bioinformatics analysis, firstly, we used FASTP (https://github.com/OpenGene/fastp; accessed on 4 June 2022) software to filter raw reads with conditions of (1) removing reads containing more than 10% of unknown nucleotides (N); (2) removing reads containing less than 80% of bases with quality (Q-value) > 20. Then, FLSAH [29] (version 1.2.11) was used to assemble the reads to tags, and QIIME [30] (version 1.9.1) pipeline was used to filter noisy sequences of raw tags. Following this, the effective tags were clustered into operational taxonomic units (OTU) of ≥97% similarity using the UPARSE [31] pipeline. The tag sequence with highest abundance was selected as representative sequence within each cluster. Then, representative OTU sequences were classified through a naïve Bayesian model using an RDP classifier [32] (version 2.2) based on the SILVA database (version 132) for bacterial taxonomy (16s rRNA metabarcoding data) [33], with a confidence threshold value of 0.8. All figures were generated using R projects. Venn analysis to compare the OTU among the different groups was performed in R using the VennDiagram package (version 1.6.16) [34]. Alpha diversity was analyzed by calculating Sob (assessed species richness level) and Shannon (comprehensively assessed richness and evenness of species) indices in QIIME [30] (version 1.9.1). Finally, a principal component analysis (PCA) was performed in R using the vegan package (version 2.5.3; http://CRAN.R-project.org/package=vegan; accessed on 4 June 2022) to assess the sample composition relation. All analyses in this section were performed using the numbers of OTU without any model transformation.

## 3. Results

### 3.1. Sugarcane Wax Composition and the Major Metabolites

**Figure 1:**
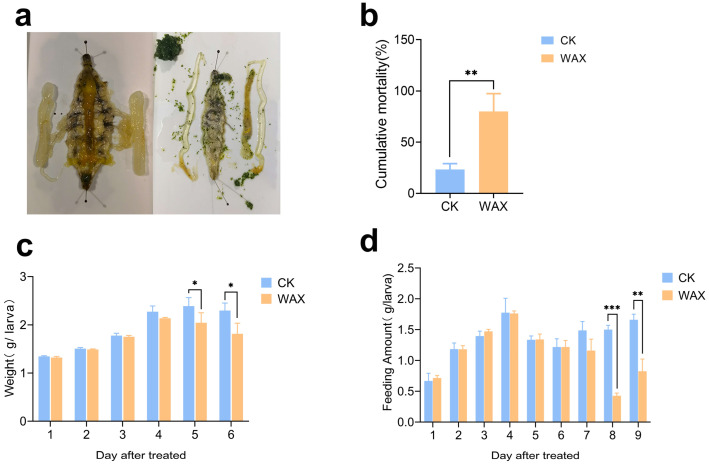
Inhibiting effects of sugarcane wax on silkworms. CK stands for normal feeding and WAX stands for wax feeding. Each treatment was replicated three times, and each replicate included 10 silkworms. (**a**) The gut of silkworms 10 days after wax feeding. (**b**) Mortality rate of silkworms fed with wax for 10 days. (**c**) Weight of silkworms fed with wax for 10 days. (**d**) Food intake of silkworms after 10 days feeding. ‘*’, ‘**’ and ‘***’ indicate *p* < 0.05, 0.01 and 0.001, respectively.

The GC-MS/MS technique was employed to assess the composition of sugarcane wax, with categories and abundance being determined based on Z-scores. A total of 157 metabolites, classified into 15 categories, were detected in wax samples. These categories included acids (6), alcohol (9), aldehyde (22), amine (4), aromatics (13), ester (28), halogenated hydrocarbons (1), heterocyclic compounds (22), hydrocarbons (23), ketone (17), phenol (2), nitrogen compounds (1), sulfur compounds (1), terpenoids (3), and others (5) (Figure 1). A thorough overview of the metabolites is provided in Supplementary Table S1.

Identification of the twenty most abundant metabolites of sugarcane wax was conducted based on peak area. These metabolites were naphthalene, isophorone, 2,4-nonadienal,(e,e), 6-methyl-3(2h)-pyridazinonem, hexanal, 2-heptenal, (z)-, 3-methylbenzothiophene, n-methylglycine, cobalt, bis(.eta.-5-piperidinylcyclopentadienyl)-, benzene, pentamethyl-, benzeneacetaldehyde, nonanal, (5r,8ar)-5-propyloctahydroindolizine, benzaldehyde, 2,5-dihydroxy-4-isopropyl-2,4,6-cycloheptatrien-1-one, 2-acetonylcyclohexanone, nonanoic acid, benzene, 1,2,3,5-tetramethyl-, benzene, 1,2,3,5-tetramethyl-, trans-4,5-epoxy-(e)-2-decenal and naphthalene, 2-methyl—(Table 1). These findings have revealed the composition of the sugarcane wax and highlighted the most abundant metabolites.

### 3.2. The Inhibited Appetite and Growth of the Silkworm Fed with Sugarcane Wax

To evaluate the insect-resistant properties of sugarcane wax, we performed a feeding trial on silkworms with the wax obtained from YT71210. After feeding wax, we observed a notable change in the internal organs of silkworms, exhibiting obvious symptoms of indigestion (Figure 1a). The mortality rate of silkworms fed with wax was significantly higher than that of the control group (Figure 1b). Moreover, it was observed that the body weight and the food intake of silkworms had decreased significantly five days and eight days after they were fed (Figure 1c,d). Taken together, our results suggested that the sugarcane wax could effectively inhibit the feeding behavior of silkworms and lead to serious growth retardation.

### 3.3. Bacterial Diversity Changed in the Gut of Silkworms Fed with Sugarcane Wax

To investigate the impact of sugarcane wax on the bacterial diversity of internal organs, we performed the 16s rRNA metabarcoding sequencing on the gut and ordure of silkworms between control and sugarcane wax feeding groups. We sampled three biological replicates for each sample vs treatment combination (*n* = 12; 2 samples × 2 treatments × 3 replicates) (Table 2). We detected 1,424,197 effective tags in the entire dataset, with an average of 118,683 tags. Overall, these effective tags represent the presence of 260 unique OTUs in all groups examined. The OTUs number ranged from 49 for GCK-3 to 197 for OWAX-2 (GCK refers to the gut samples from the control group; OCK refers to the ordure samples from the control group; GWAX refers to the gut samples from the wax feeding group; OWAX refers to the ordure samples from the wax feeding group) (Table 2).

**Figure 2:**
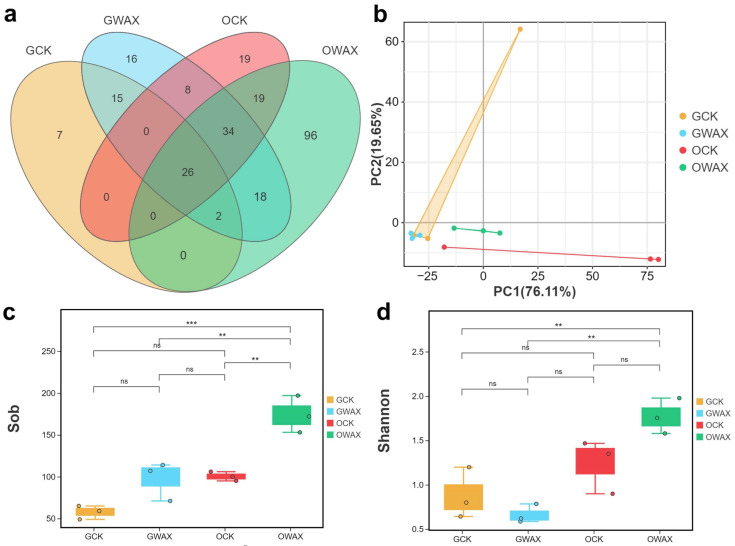
Bacterial diversity of gut and ordure for silkworms. (**a**) Venn analysis of the bacterial OTUs. (**b**) Principle components analysis based on OTUs. The abscissa represents PC1, and the ordinate represents PC2. Observed species index (**c**) and Shannon index (**d**) of OTUs. The top and bottom whiskers of boxes represent the maximum and minimum values; the line inside the box represents the median, the top margin of the box represents the upper quartile and the lower margin of the box represents the lower quartile. ‘**’ and ‘***’ indicate *p* < 0.01 and 0.001, respectively. ‘ns’ indicates non-significant.

**Figure 3:**
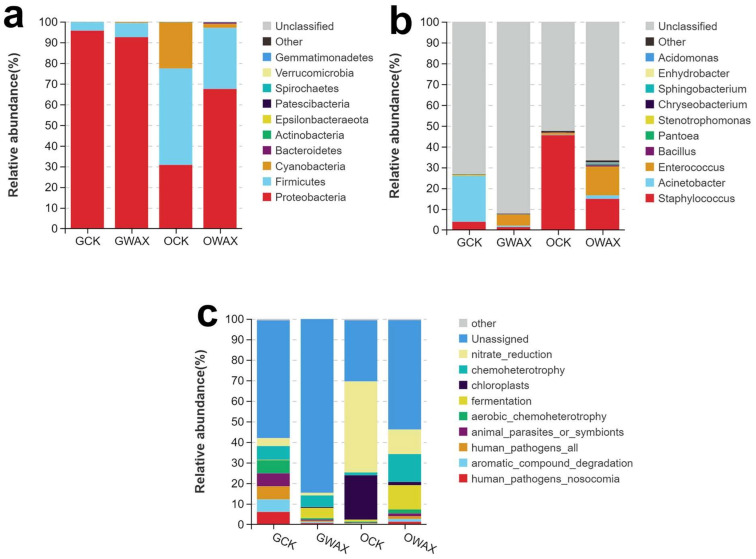
Bacterial community composition and functional annotation of gut and ordure form silkworm. Community composition analysis of bacteria in (**a**) phylum and (**b**) genus levels. The abscissa represents the group name, and the ordinate represents the relative abundance. (**c**) FAPROTAX database annotation. The different colors represent the functional group of the OTUs. The abscissa represents the group name, and the ordinate represents the relative abundance.

We further conducted an analysis of microbial diversity and functional annotation. We observed that the number of unique OTUs in the gut and ordure samples were 2.3-fold and 5.1-fold in the wax feeding group compared to the control group, respectively (GWAX = 16; GCK = 7; OWAX = 96; OCK = 19) (Figure 2a). The principle components analysis (PCA) based on bacterial OTUs abundance revealed a clear structure between the control group and the wax feeding group in ordure samples rather than gut samples (Figure 2b). To further assess species diversity, we calculated alpha diversity indices and conducted a t-test to compare the difference between samples. Sobs index and Shannon index for the gut and ordure samples ranged from 57.6 to 174 and from 0.66 to 1.77 in average for groups (Figure 2c,d). It was found that Sobs for OWAX group were significantly higher than OCK group (*p* = 0.002). In addition, we observed a notable difference in the bacterial communities based on the UNITE databases (Figure 2). This analysis suggested that *Proteobacteria* (95.66%, 92.5%, 30.75% and 67.47% in GCK, GWAX, OCK and OWAX, respectively), *Firmicutes* (4.19%, 6.83%, 46.62% and 29.51% in GCK, GWAX, OCK and OWAX, respectively) and *Cyanobacteria* (0.06%, 0.45%, 22.25% and 1.97% in GCK, GWAX, OCK and OWAX, respectively) were the top three abundant bacteria at the phylum level (Figure 3a), while *Staphylococcus* (3.92%, 1.33%, 45.51% and 14.93% in GCK, GWAX, OCK and OWAX, respectively), *Acinetobacter* (22%, 0.61%, 0.24% and 1.67% in GCK, GWAX, OCK and OWAX, respectively) and *Enterococcus* (0.17%, 5.4%, 0.85% and 13.86% in GCK, GWAX, OCK and OWAX, respectively) were the three most abundant bacteria at the genus level (Figure 3b). Interestingly, we observed obvious patterns as follows: (1) the *phylum Proteobacteria* was dominant in all samples; (2) the abundance of *Cyanobacteria* was strongly reduced in the OWAX samples; and (3) the abundance of *Enterococcus* in both gut and ordure samples was largely increased in the wax feeding group (Figure 2). Finally, a functional analysis was conducted by annotating the data with FAPROTAX database (Figure 3c). We found that the OTUs term of “fermentation” was increased in abundance by wax feeding in both gut and ordure samples (0.22%, 4.99%, 0.91% and 11.79% in GCK, GWAX, OCK and OWAX, respectively), the term of “chemoheterotrophy” showed a remarkably high abundance in the OWAX group (6.60%, 5.66%, 1.34% and 13.54% in GCK, GWAX, OCK and OWAX, respectively); the term of “nitrate_reduction” showed a decrease in wax quality after wax feeding in both gut and ordure samples (3.82%, 1.24%, 44.29% and 11.96% in GCK, GWAX, OCK and OWAX, respectively).

## 4. Discussion

Sugarcane wax is a kind of unexploited resource, and it is essential to explore its composition. Using the GC-MS/MS approach, we discovered 157 metabolites in the wax of sugarcane variety YT71210. There are some previous studies that have reported the analysis of wax metabolites with some differences in results. Inarkar (2012) reported that sugarcane wax comprised alkanes (28.83%), ester (66.26%) and fatty acids (4.58%) [35]; Asikin (2012) reported that sugarcane wax comprised 55–60% aldehyde and sterol esters and 32–40% alcohol [36]. In our result, hydrocarbons (alkanes) were the most diverse metabolite (23 of 157) but none of hydrocarbons metabolites were found in the top 20 abundant (Table 1 and Table S1). This suggests that the composition of wax has a high degree of variability. In addition, we identified 157 different wax metabolites, which is significantly more than the 25 metabolites identified in a previous study [14]. Interestingly, we found naphthalene and isophorone were the two most abundant metabolites in the wax samples, which are two metabolites reported as being associated with insect resistance in plants [29,30,31,32,33]. Naphthalene is one of the ingredients of insect repellents [37,38], while isophorone can be found in an insect attractant [39,40,41], which may suggest that metabolites isolated, purified and formulated from sugarcane wax could be used to control sugarcane borers. In addition, Wartha (2022) reported that specific sugarcane wax metabolites were associated with insect resistance of sugarcane germplasm [14]. Thus, these metabolites could be used as parameters or indicators to evaluate the ability of the sugarcane resistance to insects. Furthermore, a combined approach of gene co-expression and wax metabolomics could be used to obtain new insights into wax biosynthesis in sugarcane and to predicate the genes associated with a specific group of wax metabolites. It is of interest to explore the potential of these metabolites in insect control and in the prediction of promising sugarcane varieties with insect resistance.

In this study, we found that the sugarcane wax could significantly inhibit the feeding behavior of silkworms and lead to serious growth retardation. Revealing how sugarcane wax resists insects is beneficial to the application of wax resources. Thus, we conducted wax feeding experiments with the model insect silkworm and found that wax affects the digestive process of silkworms. Although the mulberry leaf surface also consists of wax components, their effect is mainly on water content protection and is never harmful to silkworms [42]. Therefore, the cause of death of the silkworms in the feeding group of waxed leaves resulted from certain components of sugarcane wax. There is no reference on the toxicity of sugarcane wax to silkworms; in this study, we found that the top one metabolite of sugarcane wax is naphthalene, which is an insect repellent [37,38]. It would be interesting to further investigate the effects of naphthalene on silkworm growth and development in future.

Further metabarcoding analysis showed that the intestinal microbial diversity of wax-fed silkworms was disturbed. Wax feeding of silkworms resulted in an increase in the diversity of intestinal bacterial OTUs of silkworms (Figure 2a), which also was shown to be significantly different between SOB index form groups OCK and OWAX (Figure 2c). Therefore, wax feeding might cause the unbalance of microbial diversity in the silkworm’s gut. In addition, in results of microbial taxon, multiple studies have shown that the intestinal microbial taxon of silkworms is mainly composed of *Proteobacteria* (phylum level), *Firmicutes* (phylum level) and *Enterococcus* (genus level) [19,20,43], which is basically consistent with our results. However, we found that the abundance of *Enterococcus* in the wax group (OWAX and GWAX) was significantly increased (Figure 2f), and the in FAPROTAX database annotation, the genus was classified to “fermentation” (Figure 2g), which suggests this genus has a biomass conversion function. *Enterococcus* is a kind of bacteria that specially intergrowth the gut of silkworm and has the function like synthesizing amino acids [17], and suppresses sucrose-induced hyperglycemia [18]. Meanwhile, the genus has antimicrobial activity [16], and its abundance will also could be affected by other harmful bacterial infections [44]. Therefore, the increased abundance of *Enterococcus* in our result might indicate an immune response to wax feeding of silkworm. Taken together, sugarcane wax intake affected the balance of intestinal microbiota of silkworms and had insect-resistant effects on the growth of silkworms.

## 5. Conclusions

The present study investigated the composition of sugarcane wax and identified some metabolites that could be useful in insect control. The feeding experiments with model insect silkworms demonstrated that sugarcane wax adversely affects the feeding behavior and growth of silkworms. Further analysis of the intestinal microbiome revealed that sugarcane wax has a disruptive effect on the gut microbe communities of silkworms. Our study indicated the potential of sugarcane wax as a novel natural insecticide and laid a foundation for further research on sugarcane wax.

## Figures and Tables

**Table 1:** Top 20 metabolites in the wax of sugarcane YT71210.

Compounds	Class	Formula	CAS	Peak Area
naphthalene	Aromatics	C_10_H_8_	91-20-3	3.90 × 10^6^
isophorone	Ketone	C_9_H_14_O	78-59-1	3.03 × 10^6^
2,4-nonadienal, (e,e)-	Aldehyde	C_9_H_14_O	5910-87-2	1.70 × 10^6^
6-methyl-3(2h)-pyridazinone	Heterocyclic compound	C_5_H_6_N_2_O	13327-27-0	1.11 × 10^6^
hexanal	Aldehyde	C_6_H_12_O	66-25-1	8.59 × 10^5^
2-heptenal, (z)-	Aldehyde	C_7_H_12_O	57266-86-1	8.20 × 10^5^
3-methylbenzothiophene	Heterocyclic compound	C_9_H_8_S	1455-18-1	7.78 × 10^5^
n-methylglycine	Acid	C_3_H_7_NO_2_	107-97-1	7.21 × 10^5^
cobalt, bis(.eta.-5-piperidinylcyclopentadienyl)-	Heterocyclic compound	C_20_H_28_CoN_2_	1000162-04-6	6.39 × 10^5^
benzene, pentamethyl-	Aromatics	C_11_H_16_	700-12-9	6.38 × 10^5^
benzeneacetaldehyde	Aldehyde	C_8_H_8_O	122-78-1	6.37 × 10^5^
nonanal	Aldehyde	C_9_H_18_O	124-19-6	5.69 × 10^5^
(5r,8ar)-5-propyloctahydroindolizine	Heterocyclic compound	C_11_H_21_N	120057-35-4	4.90 × 10^5^
benzaldehyde	Aldehyde	C_7_H_6_O	100-52-7	4.69 × 10^5^
2,5-dihydroxy-4-isopropyl-2,4,6-cycloheptatrien-1-one	Ketone	C_10_H_12_O_3_	54755-56-5	4.34 × 10^5^
2-acetonylcyclohexanone	Ketone	C_9_H_14_O_2_	6126-53-0	4.10 × 10^5^
nonanoic acid	Acid	C_9_H_18_O_2_	112-05-0	3.96 × 10^5^
benzene, 1,2,3,5-tetramethyl-	Aromatics	C_10_H_14_	527-53-7	3.87 × 10^5^
trans-4,5-epoxy-(e)-2-decenal	Aldehyde	C_10_H_16_O_2_	1000360-26-3	3.50 × 10^5^
naphthalene, 2-methyl-	Aromatics	C_11_H_10_	91-57-6	2.43 × 10^5^

**Table 2:** Statistics of the metabarcoding sequencing data for gut and excreta samples. GCK stands for intestinal tract from control group, GWAX stands for intestinal tract from wax-treated group, OCK stands for excreta from control group, and OWAX stands for excreta from wax-treated group.

SampleID	Tags	N90 (bp)	OTUs
GCK-1	116032	466	59
GCK-2	126819	466	65
GCK-3	120080	466	49
GWAX-1	117556	466	107
GWAX-2	127945	466	114
GWAX-3	121674	466	71
OCK-1	114870	443	106
OCK-2	123914	443	95
OCK-3	115539	466	100
OWAX-1	98036	466	153
OWAX-2	118634	466	197
OWAX-3	123098	466	172

## Data Availability

The raw metabarcoding sequencing dataset of is available in the NCBI Sequence Read Archive under BioSample accession PRJNA909462.

## Supplementary Materials

The following supporting information can be downloaded at: https://www.mdpi.com/article/10.3390/life13020286/s1; Table S1: Metabolite statistics of wax form sugarcane variety of YT71210.
